# External physical vibration lithecbole facilitating the expulsion of upper ureteric stones 1.0–2.0 cm after extracorporeal shock wave lithotripsy: a prospective randomized trial

**DOI:** 10.1007/s00240-018-1100-8

**Published:** 2018-11-28

**Authors:** Rong-zhen Tao, Qing-lai Tang, Shuang Zhou, Chun-ping Jia, Jian-lin Lv

**Affiliations:** Department of Urology, The Affiliated Jiangning Hospital with Nanjing Medical University, Nanjing, 211100 Jiangsu China

**Keywords:** External physical vibration lithecbole, Upper ureteric stones, Residual stones, Stone-free rate

## Abstract

To observe the efficacy and safety of External Physical Vibration Lithecbole (EPVL) in patients with upper ureteric stones 1.0–2.0 cm after extracorporeal shock wave lithotripsy (ESWL). A total of 271 patients with upper ureteric stones 1.0–2.0 cm were prospectively randomized into two groups. One hundred and twenty-seven cases in the treatment group accepted EPVL therapy and 144 cases as control after ESWL. The stone expulsion status and stone-free rates (SFRs) between two groups were compared at the 1st, 2nd and 4th weekends by imaging examinations. All of 271 patients were randomly assigned to two groups, of which 127 patients were included in the treatment group and 144 in the control group. EPVL was successful in assisting the discharge of stone fragments. The rate of stone expulsion at day 1 in the treatment group was significantly higher than in the control group (79.5% vs. 64.6%, *P* = 0.006). The SFRs of the 1st weekend (76.3% vs. 61.8%, *P* = 0.010), the 2nd weekend (88.2% vs. 77.1%, *P* = 0.017) and the 4th weekend (92.1% vs. 84.0%, *P* = 0.042) in the treatment group were all significantly higher than that in the control group. However, no statistical significance was found in complications between the two groups (*P* > 0.05). Furthermore, in the treatment group the patients were treated a mean 4.3 sessions of EPVL. EPVL and ESWL are ideal complementary partners in the treatment of upper ureteric stones 1.0–2.0 cm, satisfying both high SFR and low complication. This method is safe and reproducible in clinical practice, and it also needs large-scale multicenter prospective studies further to prove the above conclusions.

## Introduction

Urolithiasis, a most frequent disease in urology, is a worldwide health problem in the general population because of high morbidity and frequency of recurrence [1]. However, approximately 20% of urinary stones occur in the ureter [2, 3]. Extracorporeal shock wave lithotripsy (ESWL), developed in the 1980s with advantages such as noninvasiveness, minimal or no anesthesia, and better acceptance by patients, has been introduced as an initial option for ureteric stone treatment [4, 5]. However, problems such as stone residue and gravel fusion after ESWL are still intractable in clinical practice [6]. Raman et al. reported that 43–77% patients with asymptomatic residual stones have disease progression [7].

A number of studies have reported methods (e.g. medical expulsive therapy (MET), or discharging stones by movement or inverting position, etc.) to eliminate residual gravel and increase the stone-free rates (SFRs). In 2012, Tan et al. showed that small stone fragments can be successfully extracted by iron oxide microparticles [8]. In 2013, Shah et al. found that among 26 patients with small renal stones or residual stone fragments, 65% of the patients (17 out of 26) had the stones repositioned by ultrasonic propulsion [9]. Despite the improved efficacy brought by these methods, a new device that can assist in effective residual fragment expulsion is still needed. External physical vibration lithecbole (EPVL) is a non-invasive device designed to effectively extract the fragments. It was also shown that EPVL, if combined with reasonable and effective operating methods, could assist in stone fragment discharge after ESWL. Accordingly, we designed this prospective, randomized clinical study to evaluate the efficacy and safety of EPVL in upper ureteric stone treatment after ESWL.

## Materials and methods

### Device mechanism

The EPVL (Friend I) is a novel device developed in China and has been used at our institute since September 2016. It has a simple structure: a main oscillator held by hand and a sub-oscillator placed in the treatment bed. A multi-directional harmonic motion technology is used in this device. The lateral acceleration is achieved by a physical vibration device in the base using a harmonic vibration wave in the horizontal direction mode (power: 200 W, vibration frequency: 1300–1900 blows per minute, amplitude: 5 mm). An axial effect is then produced, inducing the upper ureteric stone to separate from the ureter. The moving space is expanded by a physical vibration device in the handle through a harmonic vibration wave in the multi-direction mode (power: 40 W, vibration frequency: 2800–3500 blows per minute, amplitude: 5 mm). Ultimately, after the position change, under the direction of the extracorporeal physical vibration machine, the upper ureteric stones are actively discharged from the ureter. All through the procedure, the position changes of the stone are monitored and observed in real time by ultrasound.

### Study design

**Fig. 1:**
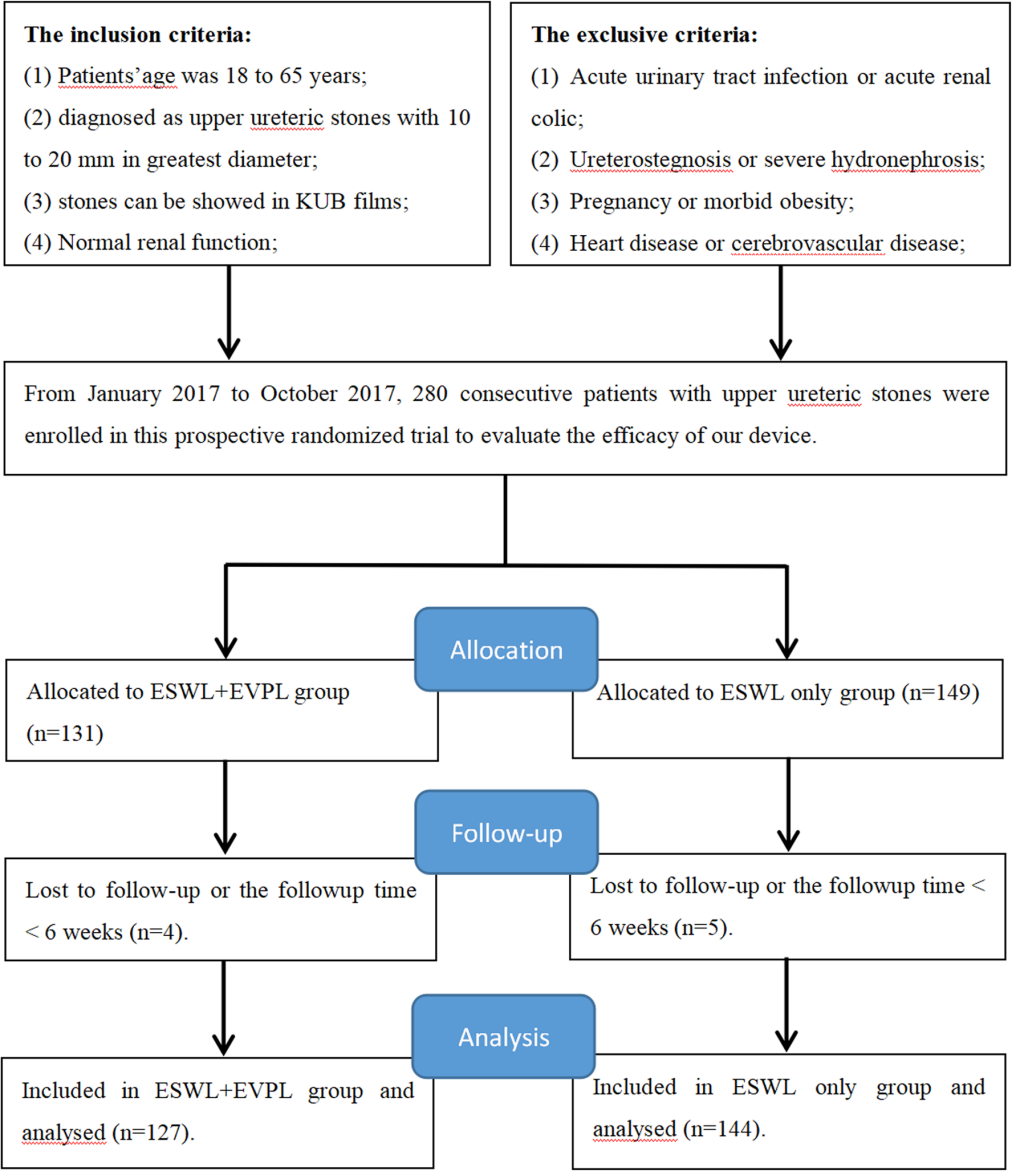
Flowchart for case selection

From January 2017 to October 2017, 271 patients with upper ureteric stones referred to our institute were recruited for this study (Fig. 1). The preoperative evaluation included medical history, physical examination, laboratory analyses (urine analysis, urine culture and/or sensitivity analysis, complete blood count, coagulation profile, blood urea nitrogen analysis, and serum creatinine levels), and radiological examinations. Patients with urinary tract infection received specific antibiotic treatment before ESWL until their urine culture turned negative. The clinical research ethics committee of the Affiliated Jiangning Hospital with Nanjing Medical University (ethics approval number: 201,700,107) approved the study protocol. The study protocol was explained to all patients and written informed consent was obtained from each patient.

After signed an informed consent, research staff at our center assigned eligible patients via a computer-generated randomization numerical code table. Random assignments were concealed in sealed envelopes.

The upper ureteric stones were initially diagnosed by abdominal ultrasound and plain abdominal X-ray for kidney, ureter, and bladder (KUB). An unenhanced computed tomography (CT) scan was performed when necessary. The maximum diameter of the stone was measured on a plain abdominal film and recorded. All procedures were performed by the same urologist. The lithotripter used in the study was an electromagnetic Dornier Compact Delta II UIMS (Dornier Medical Systems, Weßling, Germany). The stones were fragmented under fluoroscopic or ultrasound guidance. Shock waves were delivered at a fixed pulse repetition frequency of 70 SW/min. The shock wave power was gradually increased to 100% and the number of shock waves was adjusted to 2000 [10].

### Study procedure

**Fig. 2:**
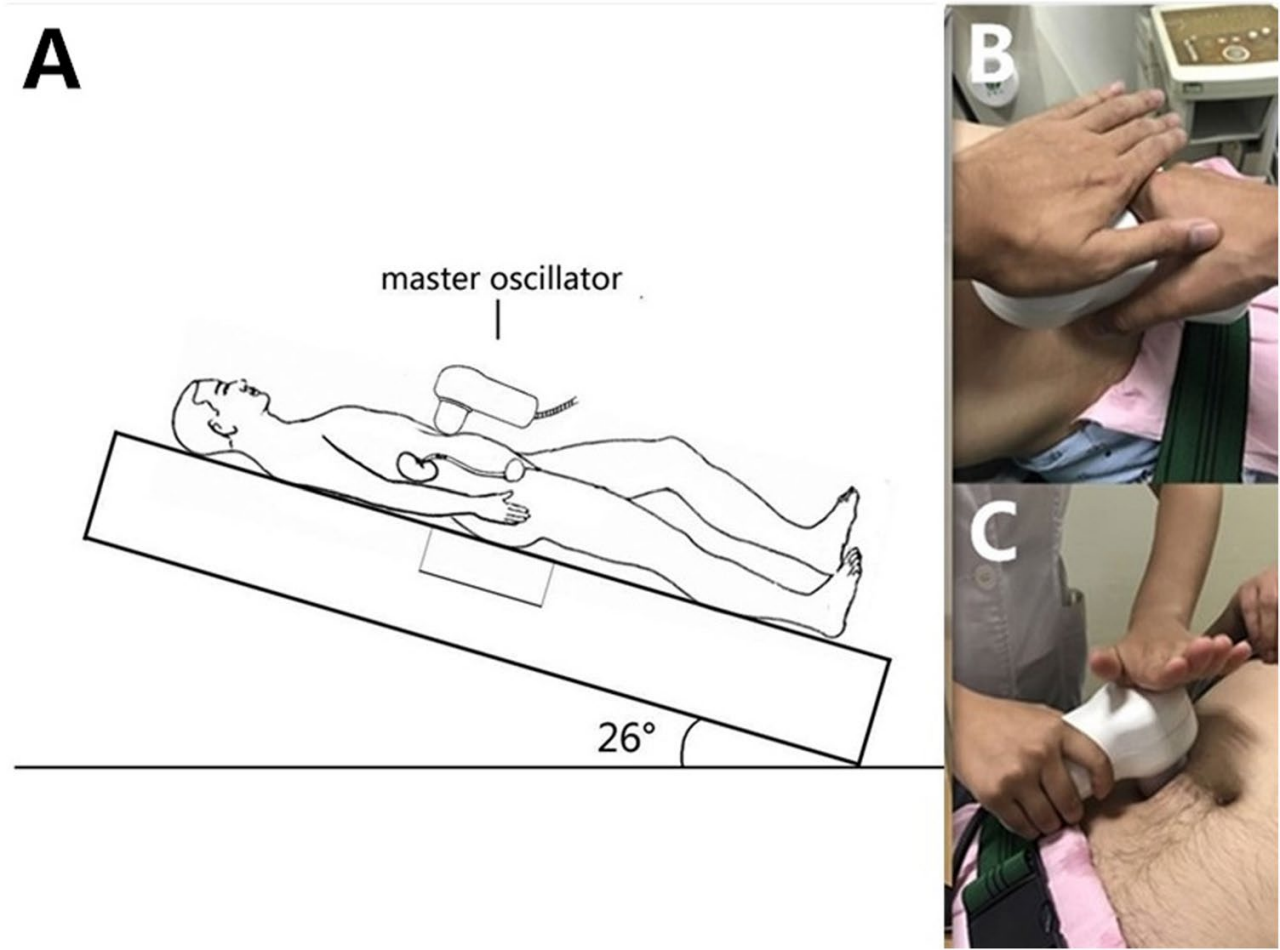
Patient in dorsal elevated position (**a**) and stone fragments discharged after EPVL (**b, c**)

In the treatment group (ESWL + EPVL), the patients underwent the first session of EPVL 30 min after ESWL without anesthesia. They were also instructed to drink 1000 mL water before the EPVL therapy. By changing the angle of the therapeutic bed, the patients were posed in the dorsal elevated position to facilitate the discharge of stone fragments (Fig. 2a). The master oscillator (vibration frequency: 2800 blows per minute; amplitude: 5 mm) was then applied over the ipsilateral ureter with pressure for 15–20 min (Fig. 2b, c). Ultrasound was used to monitor the location and movement of the ureteric stones. The next EPVL therapy would be performed in the coming weeks based on the stone expulsion outcomes.

In the control group (ESWL only), the patients were also told to drink 1000 mL water immediately after ESWL. Furthermore, the patients in both groups were required to drink at least 2500–3000 mL water daily and pass the urine through a strainer to gather possible fragments. They were also asked to complete questionnaires to report potential complications and contact the physician if any problems concerning the therapy arose. Analyses were conducted on all fragments collected. Patients with no stone expulsion at the end of the follow-up were advised to undergo another session of ESWL.

### Follow-up

No statistically significant difference was found between the two groups at the time of diagnosis or treatment. The patients in the treatment group had one to six sessions of EPVL therapy. The stone-free status at different times was considered as the primary outcome of the study. The secondary end points were complications related to EPVL therapy. The SFRs, indicating the complete absence of stone fragments, were determined using KUB film with or without ultrasonography at 3 months. Treatment failure was defined as radiologically confirmed persistence of the fragments after six EPVL sessions. The SFR, stone clearance time and complications related to EPVL were all recorded.

### Study outcomes

The primary outcome was the SFRs of upper ureteric stones at 1, 2 and 4 weeks after ESWL. Secondary outcome was the complication related to EPVL.

### Sample size and statistical analysis

We estimated that a total of 280 patients would be needed to compare a difference between groups, with a two-tailed α of 0.05 and a (1-*β*) of 0.80. Our initial estimate of sample size included an assumption of non-compliance of 10%. The statistical analysis was performed with the SPSS v.22.0 software for Windows (IBM Corp., Armonk, NY, USA). Continuous variables with normal distribution were presented as the mean ± standard deviation (SD). An independent samples *t* test was used to determine the differences in patient demographics, follow-up time, and outcomes during postoperative periods between the two groups. A chi-squared test was used to compare other clinical characteristics between the two groups. A *P* value < 0.05 was considered to be statistically significant.

## Results

In this study, 271 patients were randomly assigned to two groups: 127 in the treatment group and 144 in the control group. The patients’ demographics and clinical characteristics are shown in Table 1. The mean age of the patients at diagnosis was 49.3 years in the treatment group and 50.4 in the control group. Patients in both groups underwent one session of ESWL. No significant differences were observed between the two groups in terms of age, BMI (body mass index), gender, hypertension and diabetes histories, mean stone size, Hounsfield units, hydronephrosis, or ESWL history (All *P* > 0.05).

**Table 1:** Comparisons of patients’ demographics and clinical characteristics between two groups

Variables, mean ± SD or *n* (%)	ESWL + EPVL (*n* = 127)	ESWL only (*n* = 144)	*P* value
Age, year	49.3 ± 6.1	50.4 ± 5.7	0.126
BMI, kg/m^2^	23.6 ± 2.9	23.1 ± 3.3	0.189
Gender			
Male	83 (65.4)	96 (66.7)	–
Female	44 (34.6)	48 (33.3)	0.820
Hypertension history			
No	104 (81.9)	119 (82.6)	–
Yes	23 (18.1)	25 (17.4)	0.872
Diabetes history			
No	109 (85.8)	121 (84.0)	–
Yes	18 (14.2)	23 (16.0)	0.680
Mean stone size (mm)	1.6 ± 0.4	1.5 ± 0.5	0.073
Hounsfield units	872.3 ± 113.9	849.5 ± 133.6	0.134
Hydronephrosis			
Negative	16 (12.6)	18 (12.5)	0.981
Mild	67 (52.8)	79 (54.9)	0.729
Moderate	44 (34.6)	47 (32.6)	0.727
ESWL history			
No	113 (89.0)	125 (86.8)	–
Yes	14 (11.0)	19 (13.2)	0.586

*BMI* body mass index, *SD* standard deviation, *ESWL* extracorporeal shock wave lithotripsy, *EPVL* external physical vibration lithecbole

Differences of the clinical outcomes between the two groups are shown in Table 2. The stone expulsion rate on the first day after EPVL in the treatment group was significantly higher than that in the control group (79.5% vs. 64.6%, *P* = 0.006). In the treatment group, the SFRs in the 1st week (76.3% vs. 61.8%, *P* = 0.010), the 2nd week (88.2% vs. 77.1%, *P* = 0.017), and the 4th week (92.1% vs. 84.0%, *P* = 0.042) after treatment were all significantly higher than that in the control group. However, no statistical significance was found in manifested complications between the two groups (*P* > 0.05). Meanwhile, the patients in the treatment group were treated with a mean of 4.3 sessions of EPVL.

**Table 2:** Comparisons of clinical outcomes between two groups

Variables, mean ± SD or *n* (%)	ESWL + EPVL (*n* = 127)	ESWL only (*n* = 144)	*P* value
Stone expulsion status (day 1)			
No	26 (20.5)	51 (35.4)	–
Yes	101 (79.5)	93 (64.6)	0.006**
SFS at the 1st weekend			
No	30 (23.6)	55 (38.2)	–
Yes	97 (76.3)	89 (61.8)	0.010*
SFS at the 2nd weekend			
No	15 (11.8)	33 (22.9)	–
Yes	112 (88.2)	111 (77.1)	0.017*
SFS at the 4th weekend			
No	10 (7.9)	23 (16.0)	–
Yes	117 (92.1)	121 (84.0)	0.042*
Complications			
Dizziness	7 (5.6)	3 (2.1)	0.135
Fever	3 (2.4)	5 (3.5)	0.590
Perirenal hematoma	1 (0.8)	2 (1.4)	0.637
Mean EPVL times	4.3 ± 0.6	–	–

*SD* standard deviation, *ESWL* extracorporeal shock wave lithotripsy, *EPVL* external physical vibration lithecbole, *SFS* stone-free status

*P < 0.05, **P < 0.01

## Discussion

With a lifetime recurrence rate of about 50%, upper urinary stone poses a serious threat to public health [11, 12]. ESWL, the most widely accepted treatment for upper urinary stones < 2 cm, is minimally invasive, and has a good patient tolerance and a low complication rate [13, 14]. Although ESWL has a 60–90% success rate [15, 16], stone residues and gravel fusion after the procedure are still intractable in clinical practice, as they still have the potential to enlarge, leading to infection and obstruction of the urinary tract [17]. Therefore, the quick discharge of residual stones from the urinary tract should be deemed as a necessity after lithotripsy.

Over the past few decades, MET, which uses calcium channel blockers or alpha 1 adrenergic receptor antagonists, has been an option for active fragment expulsion [18]. However, due to the increased number of fragments after ESWL, the spontaneous stone expulsion rates decrease and renal colic recurs after MET [19]. Twenty-eight years ago, Brownlee and Netto described the inversion therapy to facilitate gravity-dependent clearance of lower caliceal stones [20, 21]. Since then, several studies have demonstrated that percussion, diuresis and inversion (PDI) therapy may be beneficial for patients with residual stones after ESWL [22, 23]. PDI therapy uses the force of gravity to assist passage of stone fragments by placing the patient in the prone Trendelenburg position and use percussion to the flank to cause vibrations in the renal system to assist in dislodgement of fragments [24].

Based on the PDI principle, a new device, which was called EPVL was manufactured in China. As with any procedure, technique is an important determinant of the effectiveness of EPVL. Compared with MET, non-invasive EPVL has multiple advantages. It has been widely used for residual stone fragment expulsion in our center since September 2016. The efficacy of residual stone discharge through EPVL has been confirmed by Chinese researchers. In 2016, Long Q. et al. reported that EPVL was effective in lower pole renal stone fragment discharge after ESWL, and it could be an adjunctive method for minimally invasive stone treatment. In his study, the SFR in the treatment group was significantly higher than the control group (76.5% vs. 48.6%, *p* < 0.01) [25]. In 2017, Wu et al. conducted a prospective, multicenter, and randomized controlled trial, using EPVL as a supplement to retrograde intrarenal surgery (RIRS). The treatment was effective in terms of stone clearance speed, SFR, and patient compliance [26]. However, there still lack reported studies showing the precise effect of EPVL treatment after ESWL therapy for upper ureteric stones 1.0–2.0 cm in size. Although, in their other study [27] they evaluated the efficacy of EPVL in patients with upper ureteric stones, the sample size was relatively small compared with our center. Therefore, we conducted this prospective, randomized study in our center. Our study revealed that stone expulsion rate on the first day after EPVL in the treatment group was significantly higher than that in the control group (79.5% vs. 64.6%, *P* = 0.006). What is noteworthy is that the variation of the SFR between the two groups was most significant (76.3% vs. 61.8%, *P* = 0.010) in the 1st postoperative week, then the difference gradually became less obvious but remained statistically significant in the 4th postoperative week (92.1% vs. 84.0%, *P* = 0.042) (Table 2). Moreover, no statistical significance was found in the occurrence of complications between the two groups (*P* > 0.05), indicating that EPVL therapy can noticeably speed up the discharge of residual stones after ESWL.

Our study proves the advantages of EPVL. First, using the rotating couch with a rotation angle of 26 degrees can easily pose the patient in a dorsal elevated position to facilitate the discharge of stone fragments. Second, under ultrasound guidance, the operator can then adjust the pressure and the depth of the master oscillator. Third, the multi-directional harmonic vibration wave produced by the physical vibration device in the handle can actively push the stone forward along the ureter and eventually expel the stones together with urine.

However, this study also has some limitations. First, the follow-up time was short, which may affect the outcomes. Second, CT was not used in all patients for the follow-up, which might be a bias for the diagnosis of residual stone. Third, the study was solely based on patients from a single center with a small sample size, which may potentially cause a certain sampling error.

## Conclusions

In conclusion, our study shows that EPVL and ESWL are ideal complementary partners with high SFRs and low complication occurrence in the treatment of upper ureteric stones of 1.0–2.0 cm size. This method is safe and reproducible in clinical practice. However, further large-scale multicenter prospective studies are needed to corroborate the above conclusions.
